# A multicentre trial of intensive immunosuppressive therapy combined with umbilical cord blood for the treatment of severe aplastic anaemia

**DOI:** 10.1007/s00277-022-04864-1

**Published:** 2022-06-04

**Authors:** Fang Zhou, Fengkui Zhang, Li Zhang, Qian Wu, Junjie Ma, Chunting Zhao, Ling Wang, Guitao Jie, Haiyan Zhang, Hao Zhang, Shunqing Wang, Qingliang Teng

**Affiliations:** 1 Department of Hematology, PLA 960Th Hospital, No. 25 Normal Road, Tianqiao District, Jinan, 250000 Shandong China; 2 Department of Hematology, Institute of Hematology and Blood Diseases Hospital, Tianjin, 300000 China; 3 Department of Hematology, Yantai Yuhuangding Hospital, Yantai, 264000 China; 4 Department of Hematology, The Affiliated Hospital of Qingdao University, Qingdao, 266000 China; 5 Department of Hematology, Qingdao Central Hospital, Qingdao, 266042 Shandong China; 6 Department of Hematology, Linyi Central Hospital, Linyi, 276000 Shandong China; 7 Department of Hematology, Linyi People’s Hospital, Linyi, 276000 Shandong China; 8 Department of Hematology, Affiliated Hospital of Jining Medical University, Jining, 610000 Sichuan China; 9 Department of Hematology, Guangzhou First People’s Hospital, Guangzhou, 510000 Guangdong China; 10 Department of Hematology, Taian City Central Hospital, Taian, 271000 Shandong China

**Keywords:** Intensive immunosuppressive therapy, Umbilical cord blood, Severe aplastic anaemia, Haematopoietic recovery

## Abstract

Immunosuppressive therapy (IST) is an effective treatment regimen for severe aplastic anaemia (SAA) patients without HLA-identical donors. This study further compared the outcomes between IST and IIST-UCB in SAA on the basis of research shown that IST combined with umbilical cord blood infusion (IIST-UCB) treated effectively. A total of 123 patients from 11 hospitals in China were enrolled. Sixty-nine patients in IIST-UCB group were treated with ATG + CsA + CTX combined with cord blood, while 54 patients in IST group with ATG + CsA. The overall remission rates (ORRs), complete remission (CR) rates and partial response (PR) rates of IIST-UCB group and IST group at 3 months were 69.67% vs 51.85% (*P* = .045), 21.74% vs 3.7% (*P* = .004) and 47.83% vs 48.15% (*P* = .972), respectively. After 6 months of treatment, they were 76.81% vs 57.41% (*P* = .022), 37.68% vs 11.11% (*P* = .001) and 39.13% vs 46.30% (*P* = .425), respectively. After 1 year of treatment, they were 85.51% vs 61.11% (*P* = .002), 59.42% vs 25.93% (*P* = .000) and 26.09% vs 35.19% (*P* = .275), respectively. The ORRs and CR rates of IIST-UCB group were both significantly higher than IST group after 3 months, 6 months and 1 year of treatment. The neutrophil granulocyte, platelet and haemoglobin recovery times of IIST-UCB group were significantly shorter than IST group. Compared with standard IST, IIST-UCB as an effective therapy for SAA patients without HLA-identical donors accelerated the haematopoietic reconstitution, resulting in higher early CR rates.

## Background

Severe aplastic anaemia (SAA) is a bone marrow failure disease characterised by dysregulated haematopoietic stem cell apoptosis and pancytopenia, causing bleeding and infection, which are the main causes of early death [1]. Presently, the treatment for SAA mainly includes allogeneic-haematopoietic stem cell transplantation (allo-HSCT), immunosuppressive therapy (IST) and supportive therapy [2–4]. Matching sibling donor haematopoietic stem cell transplantation (MSD-HSCT) is the first-line therapy for children and adolescents with SAA. Unrelated donor HSCT (UD-HSCT) with HLA matching ≥ 8/10 could be an available option [5, 6] for adult and older patients. However, the possibility of finding a matched unrelated donor is rather low and the process is time-consuming and so the SAA patients may miss their best therapeutic opportunity.

IST is composed of antithymocyte globulin and antilymphocyte globulin (ATG/ALG) combined with cyclosporine A (CsA) with an overall effective rate of 60–80%. However, 30% of patients do not respond to IST. Furthermore, the recurrence rate is up to 40%, while the effective rate of these relapsing patients is lower than 50%. In addition, some patients develop clonal diseases, like paroxysmal nocturnal haemoglobinuria and myelodysplastic syndrome, resulting in poorer prognoses [7, 8]. Therefore, it is important to develop new alternative therapies for older SAA patients and patients without HLA-identical donors.

In 1974, Kundtzon [9] found that human umbilical cord blood contained a number of haematopoietic stem/progenitor cells, mesenchymal stem/progenitor cells and cytokines that are able to stimulate haematopoietic reconstitution. Our previous study reported a clinical analysis of 36 cases of children with SAA undergoing intensive immunosuppressive therapy (ATG + CTX) combined with umbilical cord blood transfusions (IIST-UCB). The ORRs after 3 months and 1 year of treatment were 61.1% and 86.2% respectively, and the 3-year OS rate was 83.3%, which showed a better prognosis than patients undergoing standard IST [10]. Hence, we designed this multicentre trial to compare the effects of IST and IIST-UCB on the basis of aforementioned outcome.

## Methods

### Patients and study design

The trial was conducted from July 2016 to December 2018 with inclusion criteria as follows: (1) SAA and very severe aplastic anaemia (VSAA) patients diagnosed according to Camitta’s criteria [11] without sex or age limitations; (2) patients with newly diagnosed SAA and VSAA did not receive ATG or CSA for more than 6 months within 1 year; (3) no HLA-identical-related donors. The exclusion criteria included (1) congenital aplastic anaemia; (2) pregnancy or lactation; (3) participation in other clinical trials within 3 months before enrolment; (4) life-threatening conditions including respiratory failure, heart failure and liver or kidney failure; (5) aplastic anaemia caused by the treatment of other malignant tumours; (6) severe mental illness; (7) other malignant tumours; (8) serious or unmanageable infections; (9) ATG treatment within 6 months or more than 6 months of CsA treatment; and (10) serious allergies to biological agents. A total of 123 patients were enrolled with 69 received IIST-UCB therapy and 54 received standard IST.

The study was approved by the Ethics Committee of the 960 Hospital of Chinese People’s Liberation Army, and all patients signed informed consent forms. The study was conducted in accordance with the Declaration of Helsinki and has been registered on clinicaltrials.gov (No: NCT02838992).

### Treatment protocols

IIST-UCB group received rabbit ATG (r-ATG) 3 mg/kg/day × 5 days (− 6 day to − 2 day), CTX 50 mg/kg/day × 2 days (− 3 day to − 2 day), and infused cord blood haematopoietic stem cells on day 0. CsA (3 mg/kg/day) infusion was started at − 1 day lasting for 12–18 months. The dose of CsA was adjusted according to its blood concentration (maintained at 200–400 ng/mL). The UCB was provided by the Shandong Cord Blood Bank; it was ≥ 4/6 or 5/10 HLA matches and given in a single administration. The UCB was infused into the peripheral blood with number of 4.03 (1.64–13.69) × 10^7^/kg.

IST group received r-ATG 3 mg/kg/day × 5 days (− 5 day to − 1 day). An infusion of 3 mg/kg/day CsA was started at − 1 day lasting for 12–18 months. The dose of CsA was adjusted according to its blood concentration (maintained at 200–400 ng/mL).

### Supportive therapy

Condensed erythrocyte infusion was performed when haemoglobin (Hb) was < 60 g/L, and platelet infusion carried out with either < 10 × 10^9^/L or > 10 × 10^9^/L accompanied by the occurrence of bleeding or symptoms of intracranial haemorrhage. The patients were treated with granulocyte-stimulating factor (GSF) via subcutaneous injection at an absolute neutrophil count (ANC) < 1.5 × 10^9^/L and thrombopoietin (TPO) at a dose of 15,000 U/day until the platelet count was > 20 × 10^9^/L. In addition, symptomatic anti-infective treatments were administered when patients’s course was complicated by infection.

### Efficacy evaluation and engraftment detection

Haematopoietic recovery was defined as an ANC ≥ 0.5 × 10^9^/L for 3 days, Hb ≥ 60 g/L for 7 days without red blood cell transfusion and platelet count ≥ 20 × 10^9^/L for 1 week with no platelet transfusion. The CR and PR rates were evaluated after 3, 6 and 12 months of treatment according to the 2009 Guidelines for the Diagnosis and Management of Aplastic Anaemia [11]. Recurrence was defined as a decrease in routine blood indices from the original pretreatment levels of patients with therapeutic responses and efficacy lasting for at least 3 months or the recurrence of blood product dependence. However, recurrence was not deemed if a temporary slight decrease in routine blood indices caused by reducing CsA was observed and the index returned to pre-original treatment levels after using CsA. The patients would be considered nonresponsive if they died without completing 12 months treatment and still incorporating into the efficacy assessment.

Engraftment was confirmed in IIST-UCB group by short tandem repeat polymerase chain reaction (STR-PCR) at the time of granulopoietic recovery and 3 and 6 months after the start of the treatment.

### Follow-ups

The primary endpoint was the ORR 1 year after treatment. The secondary endpoints were the recovery time of the neutrophil, platelet counts and Hb, the complication rates, OS and EFS.

Day 0 means the day UCB was infused for IIST-UCB group, while the first day after r-ATG for IST group. A 1-year follow-up was required for all patients in both groups where the treatment turned out to be effective. The day of relapse or death marked the end of the follow-up if SAA or VSAA relapsed or patients died within 1 year. Blood tests and liver and kidney function were performed at least twice a week and monthly respectively before the therapeutic response occurred. Bone marrow cell morphology, cytogenetics and PNH clonal tests of peripheral blood cells via flow cytometry were performed to evaluate the efficacy and to monitor disease progression at 3, 6 and 12 months after treatment. The last follow-up was in December 2018 and median follow-up of 12 (0.5 to 12) months. The OS time refers to the duration from diagnosis to death or the last follow-up. The EFS time refers to the duration from the day of diagnosis to any event or the last follow-up. The events of interest included treatment failure, relapse, new clonal chromosomal abnormalities, PNH, MDS/AML transformation, solid tumours and death.

### Statistics

SPSS version 19.0 (SPSS Inc., Chicago, IL, USA) software was used for the statistical analyses. The data was presented as the means ± standard deviations, medians (interquartile ranges) or counts (percentages). Differences between the groups were detected by *t*-tests, Wilcoxon rank-sum tests, or chi-squared tests. The Kaplan–Meier curve analysis and the log-rank test were performed to compare the haematopoietic recovery times and OS of patients between the two groups. Univariate Cox proportional hazards regression was conducted to assess the factors affecting OS, and logistic regression analysis was performed to evaluate the factors predicting EFS.

## Results

### Clinical characteristics

The baseline clinical characteristics of the patients are shown in Table 1. There was a significant difference in age between the two groups (*P* = 0.001), but no significant difference in disease severity, sex or CsA treatment. There were 41 males and 28 females in IIST-UCB group. Among them, 52 patients were diagnosed with SAA, and 17 patients had VSAA. Twenty-four patients took CsA treatment before the treatment, and 45 patients did not take any medications. In IST group, there were 32 and 22 patients diagnosed with SAA and VSAA respectively. Fourteen patients took CsA before the treatment, while 40 patients did not take any medications. The interval from diagnosis to treatment in IIST-UCB group was significantly longer than in IST group (*P* = 0.036).

**Table 1:** The baseline clinical characteristics

Parameters	IST group (*n* = 54)	IIST-UCB group (*n* = 69)	*P*-value
Age, mean (years)	31.66 ± 16.60	21.41 ± 15.54	.001
Gender, *n *(male/female)	23/31	41/28	.064
Diagnosis to treatment, mean (months)	1.0 (0.5–12.0)	2.0 (0.5–12.0)	.036
Severity of disease, *n *(%)			.057
SAA	32 (59.26)	52 (75.36)	
VSAA	22 (40.74)	17 (24.64)	
CsA therapy, *n* (%)	14 (25.9)	24 (34.8)	.291
HLA match, *n* (%)			
4/6	-	24 (34.78)	
5/6	-	20 (28.99)	
6/6	-	2 (2.90)	
5/10	-	1 (1.45)	
6/10	-	9 (13.04)	
7/10	-	4 (5.80)	
8/10	-	7 (10.14)	
9/10	-	1 (1.45)	
10/10	-	1 (1.45)	
TNC, median (10^7^/kg)	-	3.58 (0.54–14.95)	
CD34 + , median (10^5^/kg)	-	2.25 (1.43–3.21)	

*VSAA*, very severe aplastic anaemia; *SAA*, severe aplastic anaemia; *HLA*, human leukocyte antigen; *TNC*, total nuclear cells. The data are presented as means ± standard deviations, medians (interquartile range) or counts (percentages). Comparison between the two groups was performed by *t*-tests, Wilcoxon rank-sum tests or chi-squared tests. *P* < .05 was considered significant

### Haematopoietic recovery

**Fig. 1:**
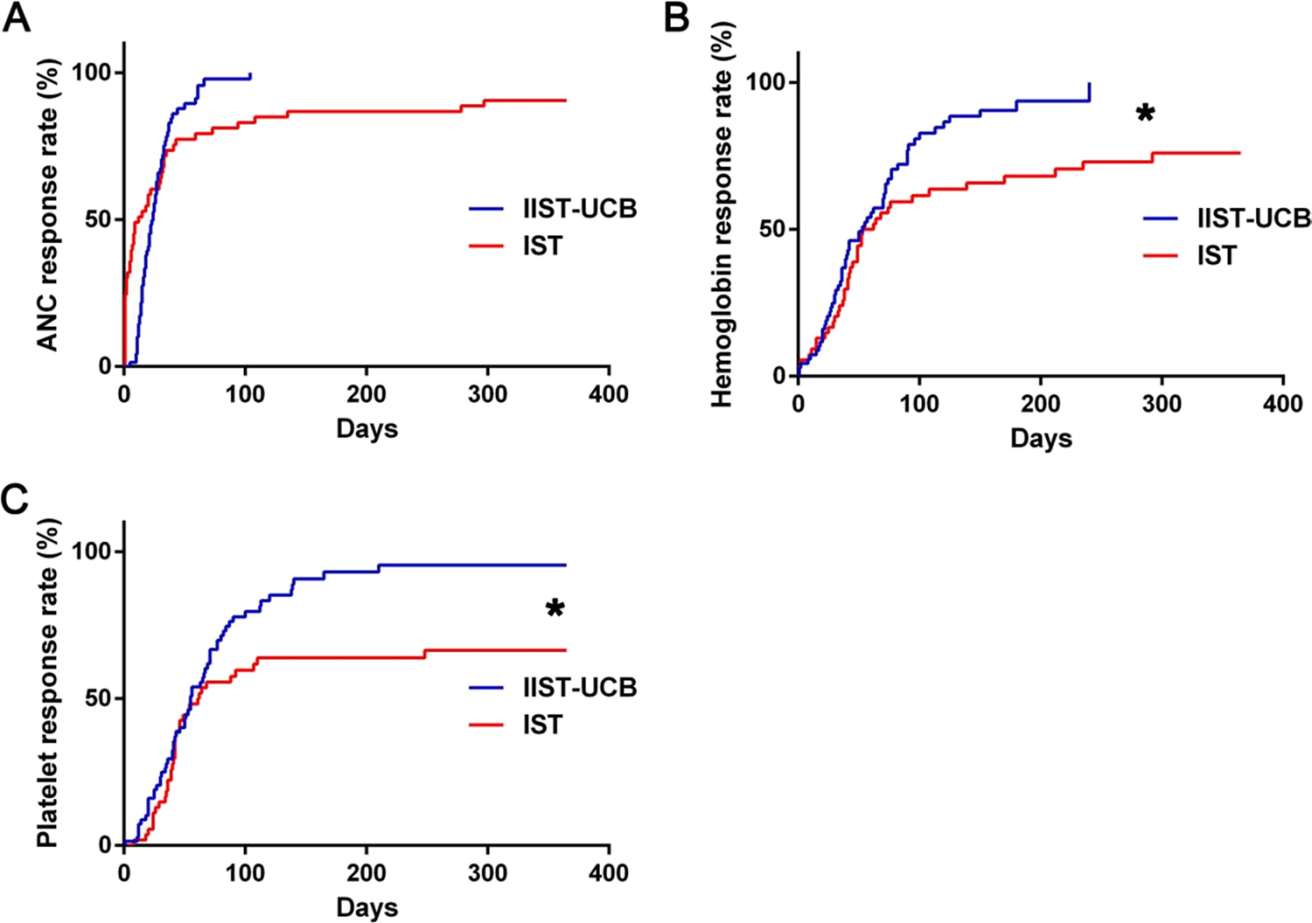
Number of days to achieve **a** ANC response, **b** HB response and **c** Platelet count response in patients. Kaplan–Meier curve analysis and log-rank tests were performed to evaluate the days required to achieve ANC, platelet and HB responses in the groups. * *P* < .05 was considered significant

The Kaplan–Meier curve analysis and log-rank test were used to further compare the recovery times of the three haematopoietic lines between two groups. Significant differences were found in the ANC recovery time between two groups (*P* = 0.035). The recovery time to Hb ≥ 60 g/L in IIST-UCB group was also significantly shorter than in IST group (*P* = 0.010). Similarly, the rate of PLT ≥ 20 × 10^9^/L in IIST-UCB group was also significantly higher than that in IST group (*P* = 0.006), as shown in Fig. 1. Significant differences were also observed in the blood transfusion volume and platelet transfusion volume between two groups. The median blood transfusion volume of IIST-UCB group was 11.0 (6.0–20.0) U, which was significantly less than that of IST group, which was 15.5 (10.0–30.25) U, *P* = 0.010. In terms of the number of platelets transfused, the median platelet volume for IIST-UCB group was 10.0 (8.0–16.0) therapeutic doses, which was significantly less than IST group, which was 16.5 (11.0–25.5) therapeutic doses, *P* < 0.001, as shown in Table 2.

**Table 2:** The transfusion volume of blood and platelets

Parameters	IST group(medians)	IIST-UCB group (medians)	*P*-value
Blood transfusion volume	15.5 (0–38)	14 (0–38)	.017
Platelet transfusion volume	16.5 (3–69)	12 (0–34)	.000

The data are presented as medians (interquartile range). The two groups were compared by the Wilcoxon rank-sum test. *P* < .05 was considered significant

### ORR, CR and PR

According to the 2009 Guidelines for the Diagnosis and Management of Aplastic Anaemia, the ORR of IIST-UCB group after treatment for 3 months was significantly higher than that of IST group (69.67% vs 51.85%, *P* = 0.045). In addition, the CR rate was higher in IIST-UCB group than in IST group (21.74% vs 3.7%, *P* = 0.004). However, there was no significant difference in the PR rate between thetwo groups (47.83% vs 48.15%, *P* = 0.972), as shown in Table 3.

**Table 3:** Clinical response after 3 and 6 months [case (%)]

Group	Cases	3-month clinical response			6-month clinical response		
		CR	PR	ORR	CR	PR	ORR
IIST-UCB	69	15(21.74)	33(47.83)	48(69.67)	26(37.68)	27(39.13)	53(76.81)
IST	54	2(3.70)	26(48.15)	28(51.85)	6(11.11)	25(46.30)	31(57.41)
*P*-value		.004	.972	.045	.001	.425	.022

The data are presented as counts (percentages). Differences between the groups were evaluated by the chi-squared test. *P* < .05 was considered significant

The ORR was still significantly higher in IIST-UCB group than in IST group (76.81% vs 57.41%, *P* = 0.022) 6 months after treatment. The CR rate of IIST-UCB group was higher than IST group (37.68% vs 11.11%, *P* = 0.001). No significant difference was observed in PR rate between two groups (39.13% vs 46.30%, *P* = 0.425), as shown in Table 3.

Twelve months after treatment, the ORR of IIST-UCB group was still significantly higher than that group (85.51% vs 61.11%, *P* = 0.002). The CR rate of IIST-UCB group was higher than IST group (59.42% vs 25.93%, *P* = 0.000). No significant difference was observed in the PR rate between two groups (26.09% vs 35.19%, *P* = 0.275), as shown in Table 4.

**Table 4:** Clinical response after 12 months [case (%)]

Parameters	IST group	IIST-UCB group	*P*-value
CR, *n* (%)	14(25.93)	41(59.42)	.000
PR, *n* (%)	19(35.19)	18(26.09)	.275
ORR (CR + PR), *n* (%)	33(61.11)	59(85.51)	.002

The data are presented as counts (percentages). Comparison between two groups was conducted by the Chi-squared test. *P* < .05 was considered significant

### Engraftment

Through STR-PCR, mixed chimerism was detected in 27 patients in IIST-UCB group (39.13%), 2 of which retained 60% of the chimerism after treatment. The other patients who responded to the treatment were ultimately tested as full recipients, indicating self-haematopoietic recovery. In addition, no significant differences in response rates in patients who did or did not have mixed chimerism after we compared those two populations in the IIST-UCB group (Table 5). The median (IQR) of CD34-positive cell count in the IIST-UCB group was 2.25 (1.43–3.21) × 10^5^/kg. The median (IQR) of CD34-positive cell count in the IIST-UCB group with mixed chimerism and without mixed chimerism was 2.46 (1.64–3.09) × 10^5^/kg and 2.21 (1.34–3.32) × 10^5^/kg, respectively. Furthermore, no significant difference was found in the CD34-positive cell count after 3, 6 and 12 months in the IIST-UCB group between patients with mixed chimerism and without mixed chimerism (Table 6)**.**

**Table 5:** Clinical response after 3, 6 and 12 months in the IIST-UCB group [case (%)]

Mixed chimerism	Cases	3-month clinical response			6-month clinical response			12-month clinical response		
		CR	PR	ORR	CR	PR	ORR	CR	PR	ORR
Positive	27	5 (18.52)	6 (22.22)	16 (59.26)	8 (29.63)	6 (22.22)	13 (48.15)	16 (59.26)	1 (3.70)	10 (37.04)
Negative	42	10 (23.81)	14 (33.33)	18 (42.86)	18 (42.86)	10 (23.81)	14 (33.33)	25 (59.52)	9 (21.43)	8 (19.05)
*P*-value		.825	.471	.279	.394	.915	.328	.989	.144	.168

The data are presented as counts (percentages). Differences between the groups were evaluated by the chi-squared test. *P* < .05 was considered significant

**Table 6:** The CD34-positive cell count after 3, 6 and 12 months in the IIST-UCB group [median(IQR)]

Mixed chimerism	Cases	3-month clinical response			6-month clinical response			12-month clinical response		
		CR	PR	ORR	CR	PR	ORR	CR	PR	ORR
Positive	27	2.46 (1.89–2.60)	2.58 (1.80–2.91)	2.51 (1.64–3.21)	2.18 (1.82–2.60)	1.90 (1.18–2.66)	2.70 (2.05–3.97)	2.44 (1.82–2.83)	1.40 (1.40–1.40)	2.68 (1.82–3.15)
Negative	42	2.89 (2.33–3.98)	2.14 (1.58–3.20)	2.01 (1.23–3.02)	2.73 (1.68–3.57)	1.67 (1.30–2.95)	2.03 (1.23–2.69)	2.25 (1.49–3.46)	1.50 (1.23–1.83)	2.44 (1.72–3.12)
*P*-value		.327	.786	.228	.336	.689	.069	.956	1	.756

The data are presented as median (IQR). Differences between the groups were evaluated by the Wilcoxon rank-sum tests. *P* < .05 was considered significant

### Safety

**Fig. 2:**
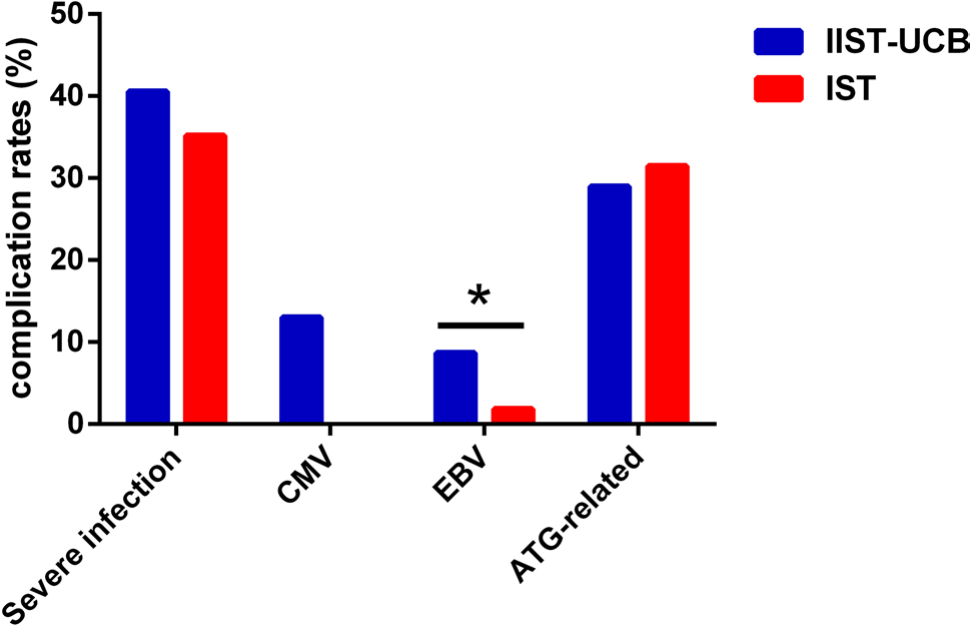
Comparison of complication rates between the two groups. Differences between two groups were evaluated by the chi-squared test. *P < .05 was considered significant

The incidence of complications in the two groups was further analysed. No significant differences were observed between the two groups in complications, which included severe infections (*P* = 0.61), cytomegaloviremia (*P* = 0.08), Epstein–Barr viremia (*P* = 0.06) and ATG-related serum diseases (*P* = 0.77), as shown in Fig. 2. Severe infection referred to infections occurred around the lung, gastrointestinal tract, urinary system and perianal abscess that were not suppressed by anti-infective treatment for > 3 days.

### Survival rate analysis and univariate and multivariate analyses of impact factors

**Fig. 3:**
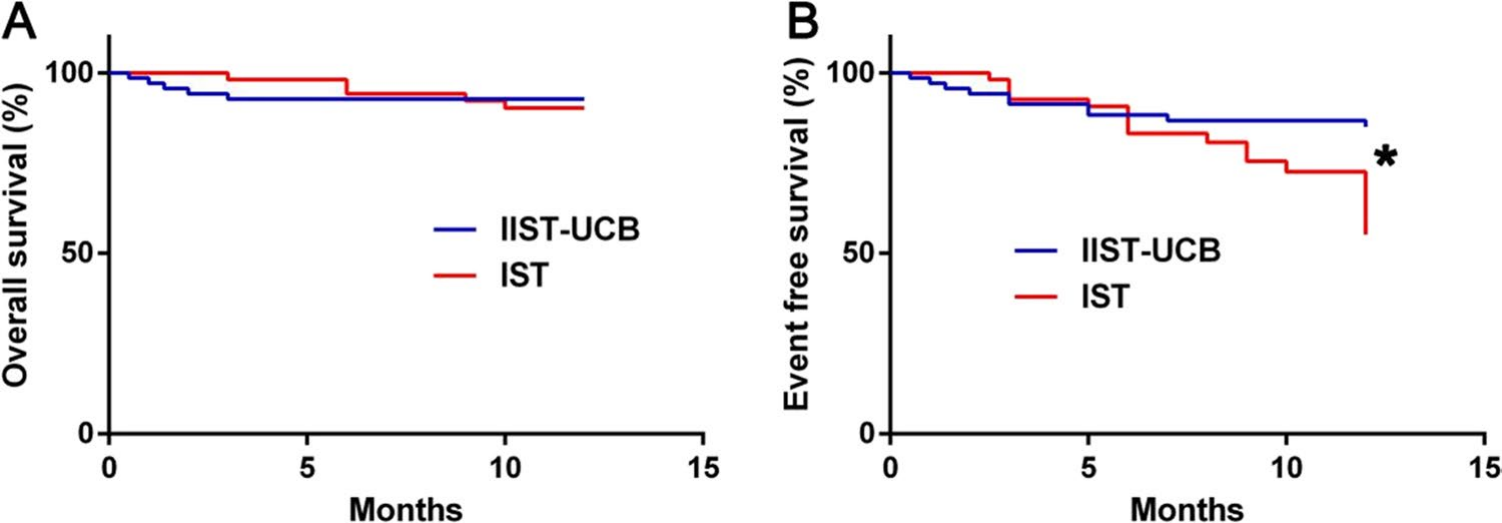
The overall survival and event-free survival between the groups. Kaplan–Meier curve analysis and log-rank tests were performed to evaluate those two groups’ survival differences. *P < .05 was considered significant

Ten of 123 patients died, five in IIST-UCB group and five in IST group, with a median of 90 (12–300) days before death. There was no significant difference in the OS rate between two groups. Regarding EFS rates, the rate was significantly higher in IIST-UCB group than in IST group (*P* = 0.006), as shown in Fig. 3. The results of univariate and multivariate analyses indicated that severe infection was an independent risk factor for patient survival (*P* < 0.05) (Table S1). Univariate analysis suggested that treatment with IIST-UCB (*P* = 0.009), blood transfusion volume (*P* = 0.000), platelet transfusion volume (*P* = 0.000) and severe infection (*P* = 0.000) were important factors affecting the EFS rate. Multivariate analysis suggested that treatment with IIST-UCB (*P* = 0.037), blood transfusion volume (*P* = 0.000) and severe infections (*P* = 0.022) were independent risk factors affecting the EFS rate, as shown in Table S2.

## Discussion

Related studies have shown that the mechanism of SAA, especially acquired or idiopathic SAA, is associated with CD34 + stem cell apoptosis caused by abnormalities in the autoimmune system and the attenuation of CD34 + stem cell differentiation to various blood cell lines [12–14]. The CD34 + cell count was low in AA who have required no treatment and have not progressed [15]. At present, the treatment of SAA with a high dose of CTX is still controversial. Some studies reported the follow-up of SAA patients taking high-dose CTX for 10 years [16] and showed 10-year overall survival rate was 88% for SAA patients treated with high-dose CTX for the first time, with an overall effective rate of 71%, indicating that high-dose CTX is highly effective for SAA. However, some studies showed that high-dose CTX reduced the risks of recurrence and transition to malignant clonal diseases delayed hemogram recovery and increased mortalities from early infections [17], declining the overall survival rate of SAA patients.

Presently, UCB is mainly used in cord blood transplantation treatment for haematological diseases [18], and few studies have investigated it as a supportive treatment for SAA patients. Xie et al. [10] reported that the overall remission rates at 3 months and 1 year after treatment were 61.1% and 86.2%, respectively, and the 3-year OS was 83.3%, which was much better than patients treated with standard IST. According to a comparison made in our centre between therapeutic treatments with ATG + CTX + UCB and HSCT, no significant differences were observed in the 2-year OS rates [19]. In some studies with small sample sizes, autologous cord blood transplantation combined with immunosuppressive agents was used for the treatment of SAA with better haematologic responses. Nonetheless, studies with larger sample sizes are still needed [20, 21]. A study of 93 SAA patients treated by IST-UCB infusion showed that the complete remission rate and overall effective rate of patients with UCB transfusion were remarkably higher than patients treated with IST 6 months after treatment [22]. Moreover, a quicker therapeutic response in ANC and platelet recovery was observed in UCB + IST group. Multivariate analysis suggested that using UCB as a supportive therapy was an independent prognostic factor for higher CR rates and ORRs. In another study including 68 SAA patients, 35 were treated with standard IST, 33 were treated with standard IST-UCB and both groups achieved the same clinical outcome [23]. All of the above studies indicated that umbilical cord blood played an important role in haematopoietic recovery of SAA patients.

In the above two studies, ATG was administered at a dose of 2.5 mg/kg/day for 5 days. In our study, the therapeutic regimens in IIST-UCB group were ATG 3 mg/kg/day × 5 days and CTX 50 mg/kg/day × 2 days to enhance the immunosuppressive effects. IIST-UCB group displayed a higher ORR and CR rate compared with IST group. IIST-UCB group had faster haematopoietic recoveries, lower blood and platelet transfusion volumes and better therapeutic effects than IST group.

These results suggested that the effects of the therapeutic regimens in IIST-UCB group were much better than those of IST. The reasons could be as follows: (1) ATG combined with CTX in immunosuppressive therapy strengthened the immunosuppressive effects, enabling full immunosuppression [24]; (2) The combination of different immunosuppressive therapies produced synergistic effects and improved the curative effects [25]; (3) UCB is rich in haematopoietic progenitor cells. Therefore, haematopoiesis can be promoted via a temporary implantation of UCB in cases of granulocyte deficiency after UCB transfusion which greatly shortens the time for haematopoietic recovery and improves the bone marrow microenvironment. When granulocyte deficiency overs, CsA can maintain normal haematopoietic function and promote haematopoiesis [26].

In addition, the therapeutic regimen in IIST-UCB group did not increase the occurrence of adverse events based on the analysis of complications and death in this study. The event-free survival rate was significantly higher in IIST-UCB group than in IST group (*P* = 0.000). A retrospective analysis of over 800 patients treated with IST showed that the 10-year transformation rate of SAA patients to MDS/PNH and other diseases was as high as 10.9% [27]. In that study, follow-up of the two groups’ patients was only carried out for 1 year, and no transformation to clonal diseases was observed. In our previous studies, long-term follow-up was conducted for SAA patients treated with IIST-UCB and no transformation to malignant clonal diseases like MDS and AML was observed.

Severe infection is an important factor in the early death of SAA patients. In this study, nine patients had multiple infections and one died of cyclosporine encephalopathy. The univariate and multivariate analyses suggested that severe infection was an independent risk factor affecting SAA patient survival (*P* < 0.05). However, there was no significant difference in the incidence of severe infection between the two groups. Although immunosuppression was strengthened by the use of immunosuppressive reagents in IIST-UCB group, there was no increase in the incidence of severe infection in IIST-UCB group which may be due to UCB. A recent study of University of Utah showed that UCB containing a factor named nNIF inhibits the formation of neutrophil extracellular traps (NETs) and is able to treat inflammation and sepsis in vitro and in infected mouse models [28]. A significant difference was observed in the OS rate of mice with sepsis between the group treated with nNIF and the group that was not indicating that UCB played an important role in IST infection. In addition, more attention should be paid to the duration of neutropenia and risk of fungal infections, after infusion high dose of CTX like 100 mg/kg.

In the past decade, with the development of transplantation technologies, alternative donor transplantation has been widely used for SAA treatment [29, 30], especially haploid HSCT [31]. Studies have shown that it has been increasingly used for children and adolescents with SAA and no significant difference in the overall survival rate of SAA patients between treating with allo-HSCT and IST. However, the EFS rate was significantly higher in allo-HSCT group than in IST group [32, 33]. Xu et al. [34] reported that the effects of allo-HSCT were also better than IST for adults. However, compared with HLA-matched HSCT, the incidence of complications like acute and chronic graft-versus-host disease (GVHD) after allo-HSCT was higher, thus adversely affecting the patients’ quality of life [35]. Engraftment detection was performed for patients in IIST-UCB group using STR-PCR in our study. Although two patients had mixed chimerism, neither of them had acute or chronic GVHD and the remaining patients showed haematopoietic self-recovery.

Although the response rate of IIST-UCB is better than that of IST, there remains a possibility that the smaller percentage of VSAA in IIST-UCB group than in IST group (24.64% vs 40.74%) may have affected the response rate. Our results may need to be verified by multicentre trial with large sample size. However, based on all the aforementioned results and the conclusion of our previous research [10], we still suggested to choose IIST-UCB as the first-line treatment compared to IST when there is no HLA-identical donor.

## Conclusion

In summary, UCB was shown to be beneficial for haematopoietic recovery, the bone marrow haematopoietic microenvironment and severe infection IST. Moreover, it showed the advantages including a lower requirement for HLA matching, the absence of related complications like GVHD, abundant sources and frequent availability when compared to HSCTUCB. Our research indicated IIST-UCB could serve as a safe and effective therapy for SAA when there is no HLA-identical donor.

## Data Availability

Not applicable.
